# Thyroidectomy with Tracheal/Cricotracheal Resection Anastomosis for Different Pathologies: Optimizing the Outcomes

**DOI:** 10.1055/s-0045-1810076

**Published:** 2025-10-09

**Authors:** Ahmed Musaad Abd-Elfattah, Ali Tawfik, Amr Hossam, Mohammed Nashaat Mohammed, Hisham Atef Ebada

**Affiliations:** 1 Department of Otorhinolaryngology, Mansoura University, Mansoura, Egypt; 2 Department of Surgical Oncology, Mansoura University, Mansoura, Egypt; 3 Department of Anesthesia and Intensive Care, Mansoura University, Mansoura, Egypt

**Keywords:** thyroidectomy, tracheal stenosis, tracheal neoplasms, thyroid neoplasms, anastomosis

## Abstract

**Introduction:**

Thyroid cancer extending to trachea has a poor prognosis. Deep tracheal invasion necessitates circumferential tracheal resection and anastomosis with thyroidectomy to achieve radical resection. Thyroid gland invasion by advanced tracheal tumors is rare. Similarly, tracheal resection with thyroidectomy is the treatment of choice for these cancers.

Neck trauma that results in simultaneous damage of a tracheal segment and thyroid gland, may necessitate tracheal resection with thyroidectomy.

**Objectives:**

The aim of this study was to evaluate the oncologic and functional outcomes in patients who had undergone thyroidectomy with tracheal/cricotracheal resection anastomosis for pathologies involving both a thyroid gland and airway.

**Methods:**

This is a study that was conducted over 5 years on 11 patients who underwent thyroidectomy with tracheal/cricotracheal resection for pathologies involving both the thyroid gland and the airway (thyroid gland tumors, primary tracheal tumors, and traumatic impaction of the thyroid gland in the trachea).

**Results:**

Successful outcomes were achieved in all patients. No intraoperative complications were reported. Minor postoperative complications were reported in 1 patient, in the form of limited surgical emphysema and air leak through the drains.

**Conclusion:**

Tracheal/cricotracheal resection anastomosis represents the best compromise between oncologic radicality and postoperative quality of life. Highly skilled teams familiar with these types of airway surgery are required to achieve optimum results.

## Introduction

The trachea lies in proximity to the thyroid gland, and it is commonly infiltrated by thyroid gland cancers. 1 From 1% to 13% of thyroid gland malignant tumors show major extracapsular extensions at diagnosis, and the incidence of tracheal invasion is up to 37%. 2 3

Tracheal infiltration is an independent risk factor for death in thyroid gland cancers. Tracheal invasion leads to a high incidence of recurrence and a mortality rate of more than 50% due to airway obstruction and severe bleeding. Accordingly, radical excision of the tumor in this situation is essential to prevent this devastating outcome. 4 5

To accomplish radical excision, a shaving procedure may be performed, however, it is adequate only if the tumor invades the tracheal perichondrium without cartilage invasion. On the other hand, deeper invasion necessitates circumferential tracheal resection anastomosis to achieve radical resection as well as good functional preservation. 6 7

The incidence of primary tracheal tumors is uncommon, accounting for only 0.09% to 0.2% of all tracheo-bronchial and lung cancers. 8 Patients always present at a late stage due to delayed diagnosis. The main symptoms are due to airway obstruction, and usually, the symptoms are misdiagnosed as chronic obstructive pulmonary diseases or bronchial asthma. Additionally, the tumor may remain symptomless until it occludes more than 75% of the airway lumen. 9 10 11

Definitive treatment of primary cervical tracheal tumors with complete surgical resection, which sometimes necessitates partial/total thyroidectomy, offers an excellent prognosis. Resection of the tumor with negative margins in the proximal and distal airway stumps, followed by end-to-end anastomosis is the curative treatment for these tumors. On the other hand, for unresectable tumors, palliative measures include tumor debulking, endobronchial radiotherapy, and airway stenting. 12

Airway injuries due to neck traumas are seen in ∼1% of all trauma patients. 13 14 Patterns of neck trauma include strangulation, clothesline neck injuries, and direct trauma due to accidents or assaults. 15 16 17 18 These injuries result in fractures in the airway cartilaginous framework, as well as mucosal tears that may lead to scarring and stenosis. 19 When there is severe tracheal injury or stenosis, tracheal resection anastomosis is a highly successful procedure entailing resection of the damaged segment and re-establishing a healthy airway. 20

The aim of this study was to evaluate the oncologic and functional outcomes in patients who have undergone thyroidectomy with cricotracheal resection anastomosis for different pathologies involving both the thyroid gland and the airway.

## Methods

This is a retrospective study that was conducted in the Otorhinolaryngology Department, Mansoura University, Egypt over 5 years (June 2018–June 2023). Reviewing data records revealed 11 patients who underwent thyroidectomy with tracheal/cricotracheal resection for pathologies involving both the thyroid gland and the airway. Informed written consents were obtained from all participants and the study was approved by the Mansoura Faculty of Medicine Institutional Research Board (R.22.11.1947).

The study included 9 patients with primary thyroid gland tumors infiltrating the trachea, 1 patient with primary tracheal adenoid cystic carcinoma infiltrating the thyroid gland, and 1 patient who sustained a clothesline neck injury which resulted in damage of the right thyroid gland lobe, and its impact inside the airway through fractured cricoid and tracheal rings. All patients of the study ( *n* = 11) underwent thyroidectomy (total thyroidectomy in 9 patients and hemithyroidectomy in 2) with single-stage tracheal/cricotracheal resection anastomosis.

### Patients with Primary Thyroid Tumors ( *n* = 9)

**Fig. 1:**
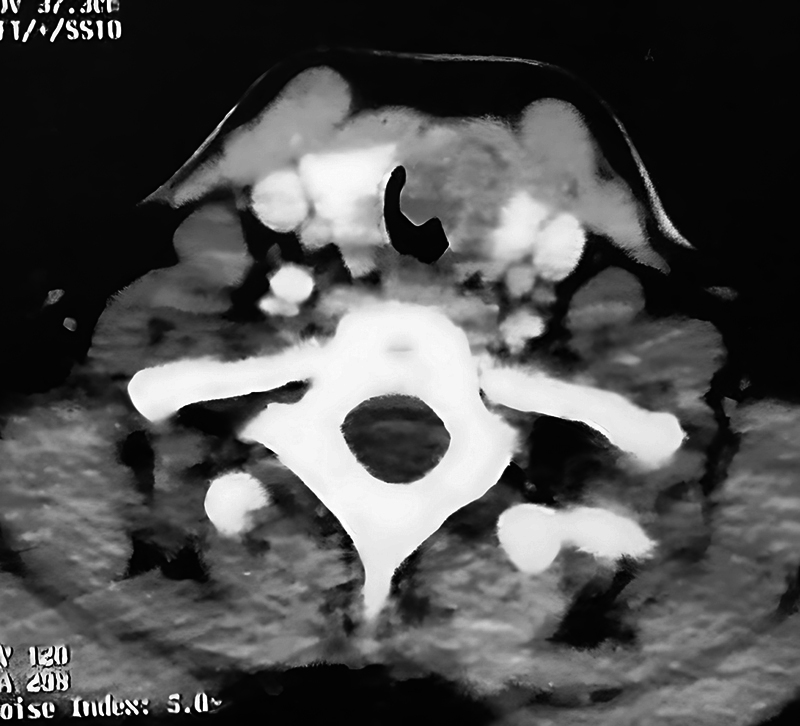
Computerized tomography (CT) scan showing a thyroid gland tumor infiltrating the trachea.

Seven out of those 9 patients presented with neck swelling (goiter). Radiological assessment was done by neck ultrasound (US), computerized tomography (CT) scan ( Fig. 1 ), and magnetic resonance imaging (MRI) when tracheal invasion was suspected. Ultrasound-guided fine needle aspiration cytology (FNAC) was performed for pathological diagnosis.

In the remaining 2 patients, the initial presentation was airway symptoms in the form of difficulty in breathing and biphasic stridor. Transnasal fiberoptic laryngoscopy revealed a normal larynx with a suspected upper tracheal mass. Neck CT and MRI were performed revealing the thyroid gland tumor with tracheal infiltration. US-guided FNAC was done for 1 patient, and on the other hand, the biopsy was obtained from the intra-tracheal tumor. This patient had severe stridor, and the decision of the airway surgery team, and the head and neck oncology team were made to perform a direct laryngo-tracheoscopy under general anesthesia for debulking of the intratracheal tumor to alleviate patient symptoms, as well as for obtaining tissue biopsy.

Shin classification 21 ( Table 1 ) was applied to categorize tracheal invasion in those 9 patients. In the current study, 6/9 patients were diagnosed as stage IV, and 3/9 patients had stage III.

**Table 1:** Shin classification

Stage	Description
I	The tumor extends through the thyroid capsule and abuts the airway external perichondrium
II	The tumor destroys the cartilage or penetrates between the tracheal rings
III	The tumor extends through the cartilage or between the tracheal rings into the lamina propria of the tracheal mucosa, without its invasion
IV	The tumor penetrates the entire thickness of the tracheal wall, presenting inside the airway with ulceration or nodules

**Fig. 2:**
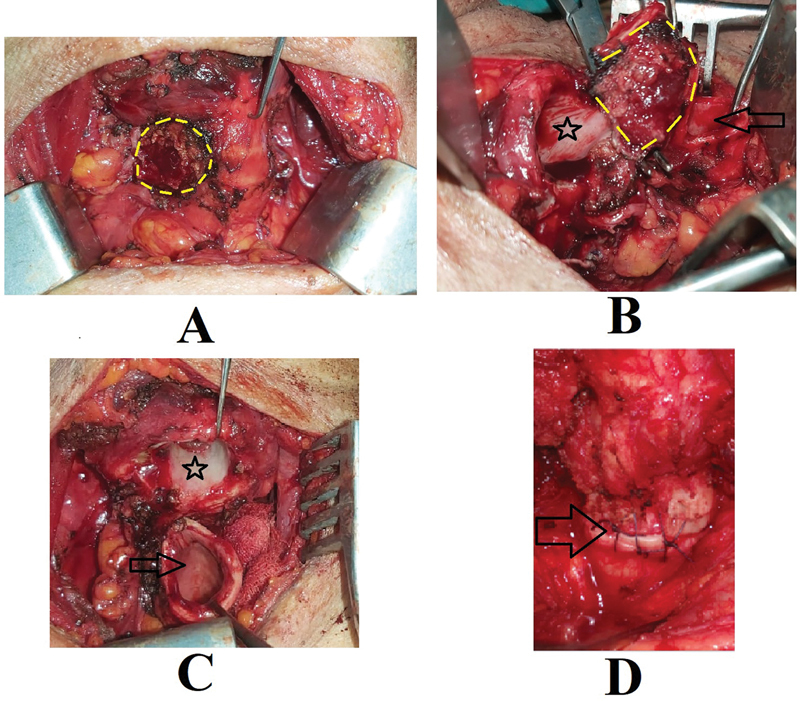
Intraoperative photos; (A): After thyroidectomy, the yellow line shows the site of invasion of the thyroid tumor inside the trachea. (B): Resection of the involved tracheal segment. The asterisk shows the proximal stump. The arrow points to the distal stump. The yellow line shows the resected segment. (C): After complete resection. The asterisk shows the proximal stump. The arrow points to the distal stump. (D): After performing the anastomosis. The arrow points to the line of anastomosis.

**Fig. 3:**
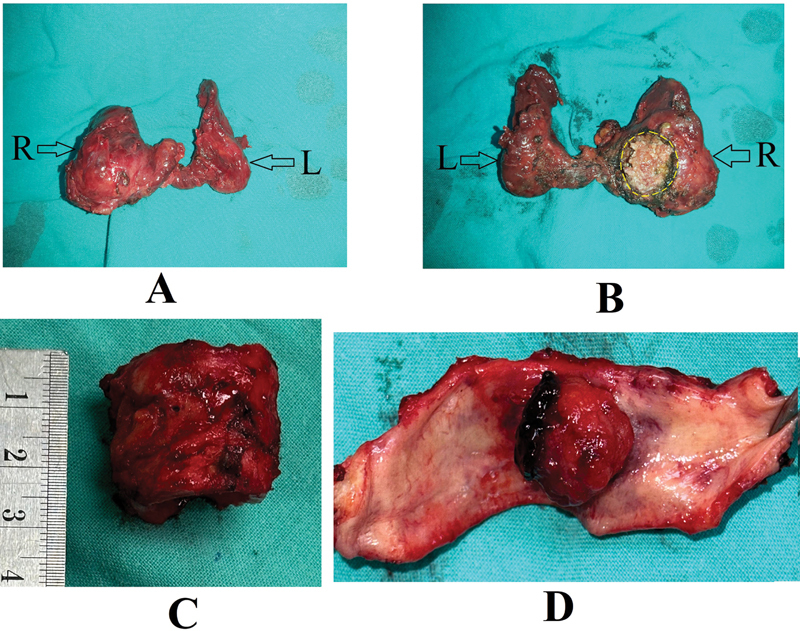
The resected thyroid gland and tracheal segment. (A): The thyroid gland. R: Right lobe. L: Left lobe. (B): The posterior aspect of the thyroid gland. The yellow dotted line shows the site of tumor invasion into the trachea. (C): The resected tracheal segment. (D): The tracheal segment is opened to show the thyroid tumor invading inside the tracheal lumen.

Total thyroidectomy with tracheal/cricotracheal resection was done in all 9 patients ( Figs. 2 and 3 ). Neck dissection was performed in 8 patients: unilateral with central compartment dissection in 6 patients, and bilateral neck dissection in 2 patients. Pathology results revealed papillary carcinoma in 7 patients and follicular carcinoma in 2 patients.

Postoperative hormonal replacement therapy, as well as radioactive iodine treatment, were prescribed by the medical oncology team. Additionally, follow-up visits were planned where appropriate laboratory and imaging studies were regularly performed.

### The Patient with a Primary Tracheal Tumor Infiltrating Thyroid Gland

A thirty-year-old female presented with dyspnea for a six-month duration. She was diagnosed with bronchial asthma and received treatment without improvement. The diagnosis of an upper tracheal mass was made on a flexible nasolaryngoscopy that was performed by an otolaryngologist. Imaging studies in the form of CT and MRI were done, showing a soft tissue mass in the upper trachea with invasion of the left thyroid gland lobe.

**Fig. 4:**
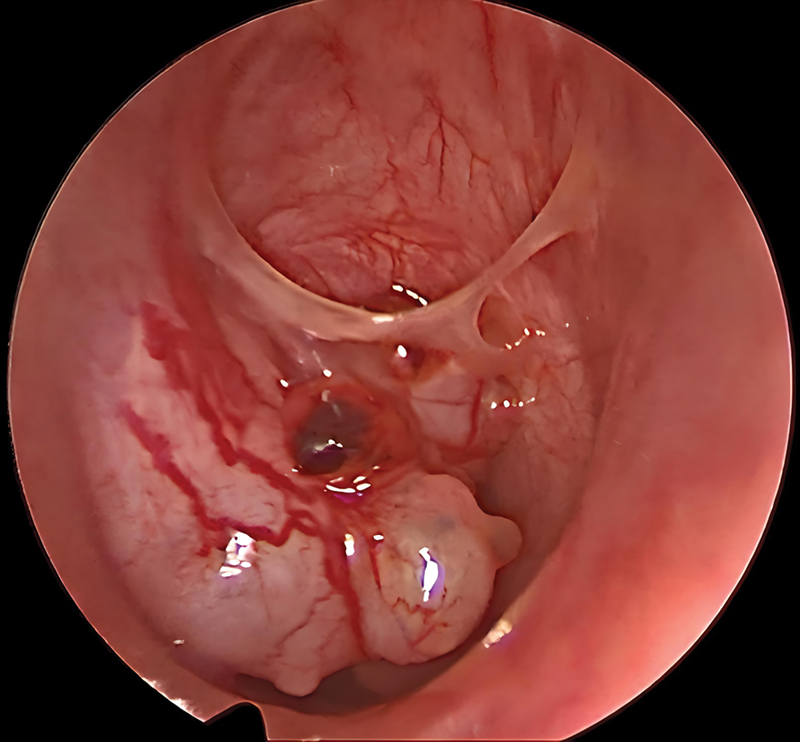
Bronchoscopic picture of a primary upper tracheal adenoid cystic carcinoma with significant airway obstruction.

Biopsy was obtained via a direct laryngobronchoscopy under general anesthesia ( Fig. 4 ), revealing an adenoid cystic carcinoma of the trachea. En-block resection of the tumor was done which entailed left hemithyroidectomy with resection of the upper three tracheal rings and the cricoid arch. The left recurrent laryngeal nerve was also sacrificed and resected for the possibility of perineural spread which is a well-known event with adenoid cystic carcinoma. Thyrotracheal anastomosis was done to re-establish the airway in one stage.

### Patient with Clothesline Neck Injury

This was a twenty-five-year-old male who presented to the emergency department with severe stridor after a clothesline neck injury. Tracheotomy under local anesthesia was performed to secure the airway, followed by neck exploration that revealed a fractured cartilaginous framework of the upper two tracheal rings and the cricoid cartilage. Interestingly, the right thyroid gland lobe was seen entrapped inside the airway through this defect.

**Fig. 5:**
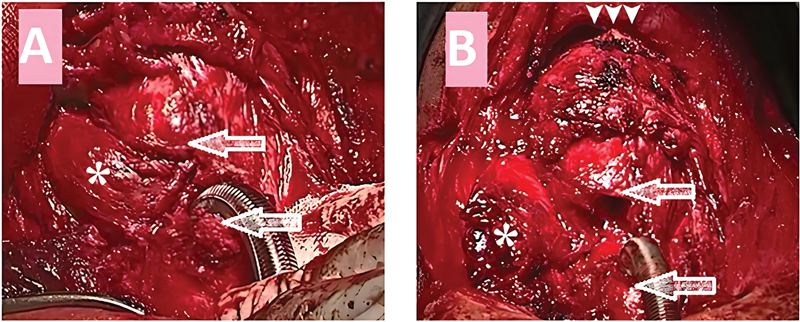
Intraoperative photos; (A) right thyroid gland lobe (the asterisk) entrapped inside the airway. The upper arrow points to the fractured cricoid cartilage and the lower arrow points to the distal tracheal segment. (B) After dissection of the thyroid lobe out of the airway. The upper arrow points to the proximal airway segment and the lower arrow points to the distal segment. The arrow heads point to the performed suprahyoid release.

The thyroid lobe was excised, and the primary closure of the airway defect was not possible. Resection of the damaged segment was done with primary thyrotracheal anastomosis ( Fig. 5 ).

In all patients of the study ( *n* = 11), the authors followed the same surgical principles of tracheal/cricotracheal resection anastomosis that was described by Grillo 22 23 and Monnier 24 25 and adopted in the authors' previous studies. 20 26 27 A cervical collar incision was planned with excision of the pre-existing scar of the previous surgery and stoma in patients with pre-existing tracheotomy. Elevation of subplatysmal flaps was then performed, upwards to the hyoid bone, and downwards to the sternum. Exposure of the thyroid gland was achieved by separation of the strap muscles in the midline. After thyroidectomy, the laryngotracheal complex was exposed. Circumferential dissection of the trachea is performed only at the level of the involved segment. Excessive dissection of the proximal and distal stumps was avoided to preserve the blood supply to improve the healing of the anastomosis.

The supra-hyoid release was the preferred release maneuver, and it was performed in all patients. It entailed the release of the hyoid bone from the attached suprahyoid muscles to decrease the tension on the anastomotic line.

Circumferential resection of the involved segment was then performed with primary end-to-end anastomosis. Tracheal resection was done in 9 patients with primary cricotracheal anastomosis ( *n* = 6) and tracheotracheal anastomosis ( *n* = 3) according to the remaining proximal and distal stumps. On the other hand, when the cricoid cartilage was involved ( *n* = 2), the cricoid arch was resected along with the involved tracheal rings (cricotracheal resection) with primary thyrotracheal anastomosis. Before proceeding with the anastomosis, biopsies of the proximal and distal stumps were obtained and sent for frozen section analysis, confirming the absence of a tumor. Negative margins were achieved in all patients with malignant lesions in the current work (10 out of 11 patients).

Single-stage procedure was done for all patients ( *n* = 11), with immediate postoperative extubation. The guardian (chin to chest) suture was not used in our patients, instead; instructions against neck extension were given for patients or patients' guardians in the first 2–3 weeks postoperatively.

## Results

Eleven patients who underwent thyroidectomy with tracheal/cricotracheal resection were included in this work. The study included 6 females (54.5%) and 5 males (45.5%). The age of the patients ranged from 25 to 62 years (mean 42 years). Single-stage surgery was done in all patients with immediate postoperative extubation. No intraoperative complications were reported in this study.

The length of the resected segment ranged from ranged from 2–5 rings (average 3.2 rings). Minor postoperative anastomotic complications were reported in 1 patient (9%), in the form of limited surgical emphysema and air leak through the drains. Conservative measures were performed until the resolution of the air leak and surgical emphysema within a few days. No major complications such as anastomotic dehiscence, excessive granulation tissue formation, or restenosis were reported in the current series.

Two patients in this series had pre-existing unilateral vocal fold paralysis. Additionally, the patient with tracheal adenoid cystic carcinoma had postoperative unilateral vocal fold paralysis after resection of the recurrent laryngeal nerve. However, those 3 patients did not experience airway compromise with a satisfactory outcome.

In the patient with upper tracheal adenoid cystic carcinoma, a Positron emission tomography (PET) scan was done 6 months after surgery and showed no evidence of recurrence. Strict follow-up of the patient was advised without the need for adjuvant radio or chemotherapy.

Follow-up period ranged from 10–70 months (mean 51 months). All patients showed successful outcomes regarding the airway condition (normal breathing, swallowing, and voice). No recurrence of the tumor was detected except in one patient who showed nodal recurrence in the contralateral neck, 8 months after the first surgery. This was managed with neck dissection and postoperative radioactive iodine.

## Discussion

Thyroid gland malignancies with tracheal invasion present with airway symptoms such as hoarseness, dyspnea, or hemoptysis. Three approaches may be used for the management of these tumors: 1) shave excision, 2) full-thick window resections with possible soft tissue flap reconstruction, or 3) segmental circumferential reresection with end-to-end anastomosis. 1 In the current study, thyroidectomy with cricotracheal resection anastomosis was the preferred approach, and it was successfully performed for all patients.

Shave resections are easy to apply, with low morbidity and no need for reconstruction. However, complete radical tumor excision is always questionable. 3 McCaffrey 28 reported that shave resections carry poorer outcomes due to residual tumors. McCarty et al 29 found that all their shaving procedures left microscopic residual disease on the trachea, and 17% of patients developed loco-regional recurrence after a mean follow-up of 81 months.

Similarly, the Window approach is less invasive than circumferential resection. 30 Nevertheless, Allen et al, 1 in their meta-analysis, reported that the pooled estimate of locoregional recurrence after circumferential resections from 19 studies was 15%. On the other hand, for window resections, the pooled incidence of locoregional recurrence, as documented in 7 studies, was 25.6%. Additionally, sputum accumulation in the airway lumen may occur secondary to the absence of pseudostratified ciliated epithelium used to close the defect after window excision. 31 Similarly, Ebihara et al 32 reported that long-term results are better with circumferential resection than with window excision. In the present work, the window approach was not preferred. Circumferential resection anastomosis was the procedure of choice.

For the thyroid surgeon who is not experienced in airway surgery, tracheal/cricotracheal resection anastomosis is usually not preferred compared with less invasive approaches such as the tracheal shave technique. This is mainly due to its potential complications. 33 34 The most feared complication is dehiscence of the airway anastomosis, with possible airway compromise, neck infections, massive bleeding, and mortality. 35 However, cooperation between the head and neck surgery team and experienced airway surgery team allows the performance of these types of surgeries with excellent outcomes and low incidence of morbidity and mortality. 20 In this study, well-trained head and neck and airway surgery teams were involved in the management of these patients and high success rates were achieved.

In a recent meta-analysis done by Allen et al 1 which included 1,179 patients with thyroid cancers invading the airway, the pooled incidence of airway complications, in the form of airway obstruction, anastomotic dehiscence, or restenosis requiring intubation or tracheotomy was 1.7%. The incidence of bilateral recurrent laryngeal nerve injury was 2.8%. For anastomotic dehiscence, the pooled estimate was 2.2%. In the present study, 1 out of 11 patients developed a minor air leak which was successfully managed conservatively. None of the patients showed major complications or required re-operation or intubation.

The incidence of anastomotic complications after tracheal/cricotracheal resection depends mainly on the length of the resected segment. Longer resections are associated with increased anastomotic tension and consequently, increased incidence of complications. 26 The maximum length of resection that permits direct anastomosis is ∼5–6 cm. which is equivalent to 7 tracheal rings. 7 30 In the current series the average length of resection was 3.2 rings (2–5 rings).

Devkota et al 36 stated that longer resections necessitate tracheal mobilization and laryngotracheal release maneuvers such as suprahyoid release, as well as maintained head flexion after surgery. In this study, suprahyoid release was routinely performed in all patients to decrease the anastomotic tension. Suprahyoid release which was first described by Montgomery in 1974 37 is safe and well tolerated. Additionally, it can be performed through the same cervical approach. The main complication of this maneuver is dysphagia and choking, 38 However, this sequelae is usually temporary and improves within a few days after surgery. None of the patients in the current series experienced choking or dysphagia.

Piazza et al 3 concluded that tracheal/cricotracheal resection for thyroid gland tumors invading the trachea is the best approach. It achieves better disease control with good functional outcomes and quality of life. In the current study, single-stage tracheal/cricotracheal resection with immediate postoperative extubation was done in all patients. Piazza et al, 3 in their study of 27 patients who underwent thyroidectomy with tracheal/cricotracheal resection for thyroid malignancies invading the trachea, reported that immediate postoperative extubation was done in 21 patients (78%). A tracheotomy was performed for 1 patient in the following days due to laryngeal edema. Another patient needed a tracheotomy due to anastomotic dehiscence. In the current series, no major complications were reported, and none of the patients required postoperative tracheotomy.

Clothesline neck injury entails a high-velocity impact on the airway after hitting a stationary object while riding a motorcycle or other vehicle. It is a fatal injury that may result in airway crushing or cricotracheal separation. 39 In the current series, the patient who sustained a clothesline injury showed an impacted right thyroid gland lobe within the fractured and severely damaged subglottic region and upper tracheal rings. A successful outcome was achieved by excision of the damaged thyroid lobe as well as the crushed airway segment that was done with primary thyrotracheal anastomosis.

Many reports of clothesline injuries exist 14 40 41 42 with a wide range of injuries to the airway (in the form of cricotracheal separation), vascular and neural structures. In all these patients, airway reconstruction with primary end-to-end anastomosis was always performed.

Regarding primary tracheal adenoid cystic carcinoma, the accepted surgical treatment is radical excision whenever possible. Adjuvant radiotherapy may be indicated especially for those with incomplete tumor excision. Furthermore, definitive radiotherapy may be used for unresectable tumors or for patients with severe comorbidities. 43 In this study, the patient with tracheal adenoid cystic carcinoma was successfully managed with radical tumor resection with cricotracheal resection and right hemithyroidectomy. Primary thyrotracheal anastomosis was performed. Adjuvant radiotherapy was not indicated.

## Conclusion

Without definitive management, primary tracheal tumors, as well as thyroid gland tumors with tracheal invasion may result in life-threatening dyspnea, and/or tracheal bleeding. Tracheal/cricotracheal resection anastomosis represents the best compromise between oncologic radicality and postoperative quality of life. Primary upper tracheal neoplasms are best managed by complete resection with end-to-end tracheal anastomosis. Highly skilled teams familiar with these types of airway surgery are required to achieve optimum results.
